# Coping strategies and resilience among patients with hypertension in Ghana

**DOI:** 10.3389/fpsyg.2022.1038346

**Published:** 2023-01-04

**Authors:** Vincent Boima, Ernest Yorke, Vincent Ganu, Anna Gyaban-Mensah, George Ekem-Ferguson, Irene Akwo Kretchy, Charles Christopher Mate-Kole

**Affiliations:** ^1^Department of Medicine and Therapeutics, University of Ghana Medical School, College of Health Sciences, University of Ghana, Accra, Ghana; ^2^Department of Medicine and Therapeutics, Korle-Bu Teaching Hospital, Accra, Ghana; ^3^Department of Psychology/Center for Ageing Studies, College of Humanities, University of Ghana, Accra, Ghana; ^4^Department of Psychiatry, Korle-Bu Teaching Hospital, Korle-Bu, Ghana; ^5^Department of Pharmacy Practice and Clinical Pharmacy, School of Pharmacy, College of Health Sciences, University of Ghana, Legon, Ghana

**Keywords:** coping strategies, resilience, hypertension, LMIC, cognitive debriefing

## Abstract

**Background:**

Hypertension is associated with high morbidity and mortality and this has been linked to poor treatment and control rates. To optimize drug treatment, patient-centered strategies such as coping, resilience, and adherence to medication may improve control rates and decrease the morbidity and mortality associated with hypertension. This study, therefore, assessed coping skills and resilience among patients with hypertension in Ghana.

**Methods:**

A cross-sectional study was conducted at Korle Bu Teaching Hospital. 224 consented patients with a diagnosis of hypertension were consecutively selected from the outpatient clinic. Questionnaires comprising socio-demographic characteristics, clinical parameters, Adult Resilience Measure, and the Africultural Coping Systems Inventory were administered. Data were analyzed using Stata version 16.1 and significance level was set at *p*-value of ≤ 0.05.

**Results:**

The mean age of participants was 62.03 ± 11.40 years and the majority were female (63%). The overall coping strategy mean score was 43.13 ± 13.57. For resilience, median relational and personal resilience (PR) scores were 32 (IQR-7) and 39 (IQR-9), respectively. Increased systolic BP significantly increases the overall coping strategy score. Collective coping strategy and systolic BP significantly increased coping scores (95%CI = 0.05–3.69 vs. 95%CI = 0.58–5.31). Overall coping strategy significantly increased personal and relational resilience (RR) domain scores by 0.004 (95%CI = 0.002–0.01) and 0.005 (95%CI = 0.003–0.006) units, respectively. This study demonstrated that Cognitive and emotional debriefing coping strategy was mostly used by patients with hypertension.

**Conclusion:**

Coping strategies had a positive and significant correlation with personal and RR, specifically collective and cognitive debriefing had a significant positive association with resilience among study participants. There is a need to actively put in measures that can improve the coping strategies and resilience among patients with hypertension to adjust to the long-term nature of the illness and treatment as this will promote better treatment outcomes.

## Introduction

Hypertension is a major public health problem with significantly associated morbidity and mortality. Globally, an estimated 1.28 billion adults aged between 30 and 79 years are living with hypertension and two-thirds live in low- and middle-income countries (LMIC) (Dzau et al., 2015). Africans have a high prevalence of hypertension with up to 50% of adults having high blood pressure (Seedat, 2015). The estimated number of adults living with hypertension in sub-Saharan Africa was 74.7 million (Seedat, 2015) with an estimated prevalence of 27% in Ghana (Bosu and Bosu, 2021).

Hypertension has been associated with 7.5 million deaths worldwide representing 12.8% of all total deaths. This accounts for 57 million disability-adjusted life years (DALYs) (World Health Organization, 2012). High blood pressure is a risk factor for coronary heart disease, stroke, chronic kidney disease, loss of vision, and peripheral artery disease (Kretchy et al., 2014). The majority of patients attend hospitals frequently on account of these complications and uncontrolled hypertension (Kretchy et al., 2014). In addition, these patients are routinely on multiple medications and are at risk of being hospitalized due to uncontrolled hypertension and treatment of these associated complications (Kretchy et al., 2014). As result, there is huge financial burden associated with the management of patients with hypertension as healthcare cost is mostly a responsibility of the affected individual in Ghana. These factors in addition to the chronic nature of hypertension, increases their risk of developing mental health problems.

Chronic disease and their treatments have been shown to cause psychological problems such as depression, anxiety, and cognitive dysfunction (Awuah et al., 2019; Amankwah-Poku et al., 2020). The prevalence of depression among hypertensive cohorts in Ghana and Nigeria was 41.7 and 26.6%, respectively (Ademola et al., 2019). A study in Nepal showed that 84% and 10.3% of patients with mild and severe hypertension, respectively, had symptoms of anxiety (Shah et al., 2022). Again, hypertension is a common risk factor for vascular cognitive impairment and Alzheimer’s disease and both collectively explain 85% of cases of dementia (Arvanitakis et al., 2019). These psychological problems affect the already burdened healthcare system and have implications for psychosocial and economic dimensions. Managing hypertension and its associated negative effects align with the Sustainable Development Goals (SDG) 3 that seek to promote health and wellbeing for all persons at all ages. These diseases are a source of stress as they impact negatively on welfare, physical integrity, future plans, and financial stability. In addition, they undermine patients’ ability to fulfill social, family and professional obligations (Livneh and Antonak, 2005). Patients’ with chronic illness encounter new situations where the usual mechanisms of resistance may not be adequate, thus, the need for additional ways of coping and living with their health conditions. As a result, there is a need to consider strategies that can improve treatment outcomes. These may include patient-centered strategies such as coping and resilience. Previous studies demonstrated a relationship between coping skills and hypertension with positive outcomes. In addition, studies have shown that resilience has an impact on patients with chronic diseases such as diabetes and hypertension amongst others (Mota et al., 2006; Cal and Santiago, 2013).

To our knowledge, there is limited research on the impact of resilience and coping in patients with hypertension in Ghana (Dzau et al., 2015; Seedat, 2015). Examining the coping strategies and level of resilience in persons with hypertension will help to educate patients on coping skills that may produce effective treatment outcomes in hypertensive patients.

## Materials and methods

This was a cross-sectional study involving 224 consecutively selected patients with hypertension, 18 years and older who attend two outpatient clinics at the Korle-Bu Teaching Hospital. Consent was obtained from the patients after they were given appropriate information about the study. Inclusion criteria comprised; patients diagnosed with hypertension who have been on medication for at least 6 months, while those with dementia, neuropsychiatric illness and a history of trauma based on information in their medical records, were excluded.

The investigators and trained research assistants collected the data. A structured questionnaire was administered to consented participants to obtain data on the socio-demographics and clinical parameters. The socio-demographic characteristics included age, gender, religion, educational status, employment status, monthly income, marital status, and socio-economic status. The clinical parameters were blood pressure readings, height, weight, and waist circumference. The standardized instruments the Resilience Scale for Adults and the Africultural Coping Systems Inventory were used to measure resilience and coping strategy of participants, respectively.

A digital blood pressure monitor (OMRON HEM-907) was used to measure blood pressure. Blood pressure was measured 3 times at least 5 min apart. The mean of the last two measures was recorded.

Anthropometric parameters such as height (m), and weight (kg) were measured and recorded. Height was measured using a stadiometer with patients wearing no shoes and recorded to the nearest centimeter. Weight was measured using a SECA Digital weighing scale to the nearest 0.5 kg. Body mass index (BMI) (30 kg/m^2^) was calculated from the weight and height. Waist circumference was measured using a tape measure and recorded to the nearest centimeter.

The revised Adult Resilience Measure (ARM-R) is a self-report measure of social and ecological resilience and is used by researchers and practitioners worldwide. It consists of 17-items and is scored 5- point Likert scale and responses include “Not at all,” “A little,” “Somewhat,” “Quite a bit,” and “A lot.” The items in the measures are all positively worded and therefore scoring involves summing of the responses obtained. In addition to overall score, scores can be obtained for two subscales [personal and relational resilience (RR)]. RR refers to characteristics associated with relationships shared with either partner or family while PR relates to intrapersonal and interpersonal items. Cronbach’s alpha is 0.82 for personal and RR subscales, and 0.87 for overall resilience subscale (Jefferies et al., 2018; Resilience Research Centre, 2018).

The 30-item Africultural Coping Scale Inventory (ACSI) was used to measure four dimensions of culture-specific coping: (a) Cognitive and Emotional Debriefing (11 items); (b) Collective Coping (8 items); (c) Spiritual-Centered Coping (8 items); and (d) Ritual-Centered Coping (3 items) (Bosu and Bosu, 2021). As part of the ACSI, participants were asked to describe a stressful situation from the past week using a 4-point Likert-type scale (0 = *did not use*, 1 = *used a little*, 2 = *used a lot*, 3 = *used a great deal*) to denote strategies they used to cope. For each subscale, items were summed, and higher scores indicated more use of that coping strategy. Cronbach’s alpha for the total scale was 0.90 (Utsey et al., 2000).

### Data analysis

The data were analyzed using Stata version 16.1. Two independent analytical processes were conducted to achieve the objective of the study; these involved descriptive and inferential analysis. For descriptive analysis, sociodemographic characteristics were summarized in tables by reporting the proportion and means of the variables involved in the study. For inferential analysis, two approaches were adopted: mean differences and regression analysis. The mean difference of the study outcomes by the explanatory variables was adopted and one sample Kolmogorov–Smirnov test was performed to assess normality. All variables did not violate the normality assumptions and were therefore confirmed using the Shapiro-Wilk test of normality. The *t*-test and ANOVA was used depending on the levels of the categorical variable. To assess factors associated with the exposure variable, authors adopted Ordinary Least Square (OLS) regression methods to assess the strength of the association by considering adjusted variables. Before OLS analysis, multicollinearity assumption was tested using the variance inflation factor where the “help from family” was highly correlated with help from friends, therefore the variable was modeled during the adjusted OLS regression analysis.

To assess the relationship between the exposure and the outcome variable, OLS was performed and adjusted with significant factors associated with the domain of the exposure variable. Analysis was performed using Stata 16.1 and the significance level was set at *p*-value < 0.05.

## Results

The study involved 224 persons living with hypertension aged between 27 and 92 years with mean ± Standard deviation of 62.03 ± 11.40 years and the corresponding median (interquartile range) was 63 (15.5) years. The proportion of female and male participants were 63 and 37%, respectively. The majority of the participants were obesed (48.9%) (Table 1).

**TABLE 1:** Demographic characteristics and the mean difference of coping strategies among participants.

Variable	Frequency	Coping strategies				
		Spiritual	Collective	Ritual centered	Cognitive/emotional	Overall
	
	*n* (%)	Mean [95%CI] ± SD	Mean [95%CI] ± SD	Mean [95%CI] ± SD	Mean [95%CI] ± SD	Mean [95%CI] ± SD
Overall		14.36 [13.68–15.04] ± 5.14	10.09 [9.41–10.77] ± 5.18	0.28 [0.15–0.40] ± 0.96	18.40 [17.56–19.24] ± 6.32	43.13 [41.34–44.92] ± 13.57
**Age**						
≤49	28 (12.5)	15.14 [13.15–17.13] ± 5.35	10.29 [8.17–12.40] ± 5.69	0.32 [−0.045-0.69] ± 0.98	16 [13.22–18.77] ± 7.44	41.75 [36.36–47.13] ± 14.47
50–59	57 (25.5)	13.91 [12.56–15.27] ± 5.20	9.91 [8.50–11.32] ± 5.41	0.19 [−0.04-0.42] ± 0.88	19.11 [17.52–20.69] ± 6.08	43.12 [39.55–46.69] ± 13.68
60–69	83 (37.0)	14.62 [13.52–15.73] ± 5.09	10.08 [8.95–11.23] ± 5.29	0.39 [0.14–0.63] ± 1.14	19.06 [17.70–20.42] ± 6.28	44.15 [41.17–47.14] ± 13.82
70 +	56 (25.0)	14.04 [12.68–15.39] ± 5.11	10.18 [8.95–11.42] ± 4.64	0.18 [−0.01-0.37] ± 0.72	17.91 [16.36–19.45] ± 5.81	42.31 [38.89–45.72] ± 12.84
Test statistic		0.50 (0.680)	0.04 (0.989)	0.70 (0.556)	2.02 (0.118)	0.32 (0.8114)
**Sex**						
Male	83 (37.05)	13.41 [12.19–14.64] ± 5.57	9.83 [8.59–11.07] ± 5.65	0.39 [0.14–0.65] ± 1.16	18.54 [17.20–19.87] ± 0.67	42.17 [38.98–45.35] ± 14.5
Female	141 (62.95)	14.91 [14.11–15.72] ± 4.81	10.24 [9.42–11.06] ± 4.91	0.21 [0.08-0.35] ± 0.82	18.33 [17.25–19.41] ± 0.55	43.69 [41.52–45.86] ± 13.02
Test statistic		−2.12 (0.035)	−0.57 (0.568)	1.33 (0.184)	0.2391 (0.8113)	−0.8082 (0.419)
**Marital status**						
Married	133 (59.64)	14.37 [13.45–15.28] ± 5.35	10.30 [9.41–11.19] ± 5.21	0.30 [0.14–0.46] ± 0.95	18.77 [17.73–19.81] ± 6.08	43.74 [41.47–45.99] ± 13.21
Single	14 (6.28)	12.64 [9.63–15.66] ± 5.72	8.71 [5.86–11.57] ± 5.41		16.35 [12.37–20.34] ± 7.57	37.71 [29.34–46.08] ± 15.88
Widowed	57 (25.56)	15.37 [14.29–16.44] ± 4.09	10.74 [9.39–12.09] ± 5.17	0.39 [0.07–0.70] ± 1.21	18.97 [12.81–18.56] ± 6.38	45.45 [42.07–48.84] ± 48.84
Divorced	19 (8.25)	12.58 [10.04–15.12] ± 5.61	7.68 [5.72–9.65] ± 4.35		15.68 [12.80–18.56] ± 6.37	35.94 [29.74–42.15] ± 13.71
Test statistic		2.04 (0.109)	2.09 (0.102)	1.19 (0.315)	1.98 (0.1174)	3.26 (0.0223)
**Educational level**						
No formal education	20 (8.93)	13.15 [11.05–15.25] ± 4.76	7.7 [5.48–9.93] ± 5.05	0.3 [−0.02-0.62] ± 0.73	16.7 [13.70–19.69] ± 6.79	37.85 [31.74–43.95] ± 13.84
Primary/JHS	55 (24.55)	15.18 [13.96–16.41] ± 4.60	10.76 [9.46–12.07] ± 4.92	0.33 [0.00–0.65] ± 1.22	17.67 [15.71–19.63] ± 7.38	43.94 [40.24–47.26] ± 13.94
SHS/vocational	91 (40.63)	14.33 [13.24–15.42] ± 5.25	10.19 [9.05–11.34] ± 5.53	0.33 [0.12–0.54] ± 1.03	19.64 [18.52–20.76] ± 5.44	44.49 [41.72–47.26] ± 13.40
Tertiary	58 (25.89)	14.05 [12.59–15.51] ± 5.58	10.11 [8.86–11.35] ± 4.78	0.14 [−0.003-0.28] ± 0.55	17.74 [16.12–19.35] ± 6.18	42.04 [38.59–45.47] ± 13.19
Test statistic		0.91 (0.439)	1.76 (0.156)	0.53 (0.665)	2.13 (0.0976)	1.52 (0.2111)
**Employment**						
Full time	80 (35.71)	14.69 [13.57–15.80] ± 5.07	10.6 [9.48–11.72] ± 5.09	0.31 [0.08–0.54] ± 1.05	19.36 [17.87–20.85] ± 6.77	44.96 [41.95–47.96] ± 13.63
Unemployed	63 (28.13)	14.63 [13.32–15.95] ± 5.31	10.22 [8.81–11.64] ± 5.69	0.25 [−0.002-0.51] ± 1.03	17.81 [16.21–19.41] ± 6.44	42.92 [39.26–46.57] ± 14.71
Retired	81 (36.16)	13.83 [12.70–14.95] ± 5.12	9.47 [8.41–10.55] ± 4.86	0.26 [0.09–0.44] ± 0.81	17.91 [16.66–19.17] ± 5.69	41.48 [38.73–44.22] ± 12.47
Test statistic		0.68 (0.506)	0.97 (0.381)	0.08 (0.9221)	1.45 (0.2378)	1.34 (0.2650)
**Monthly income**						
Less than 500	18 (25.00)	13.78 [11.62–15.93] ± 4.58	10.33 [8.04–12.65] ± 4.94	0.17 [−0.07-0.41] ± 0.52	20.72 [17.79–23.66] ± 6.25	45 [39.07–50.96] ± 12.61
500–900	20 (27.78)	13.25 [11.47–15.03] ± 3.99	9.55 [7.76–11.33] ± 4.01	0.3 [−0.14-0.74] ± 0.98	18.35 [15.23–21.47] ± 7.01	41.45 [36.19–46.71] ± 11.79
1,000–1,999	15 (20.83)	15.53 [12.60–18.46] ± 5.69	11.2 [8.97–13.43] ± 4.33		19 [15.27–22.73] ± 7.24	45.73 [38.78–52.69] ± 13.50
2,000 +	19 (26.39)	15.16 [12.51–17.81] ± 5.79	9.63 [7.46–11.81] ± 4.75	0.89 [0.09–1.7] ± 1.76	19.79 [16.68–22.89] ± 6.79	45.47 [38.55–52.39] ±
Test statistic		0.84 (0.478)	0.48 (0.698)	2.35 (0.0805)	0.42 (0.7393)	0.43 (0.7319)
**Religion**						
Christian	212 (95.50)	14.34 [13.65–15.03] ± 5.12	10.08 [9.39–10.77] ± 5.08	0.25 [0.13–0.38] ± 0.92	18.36 [17.50–19.22] ± 6.36	43.04 [41.22–44.85] ± 13.44
Islam	10 (4.50)	14 [10.01–17.99] ± 5.58	9.5 [9.37–10.74] ± 5.17	0.5 [−0.27-1.27] ± 1.08	19.1 [14.69–23.50] ± 6.16	43.04 [41.25–44.83] ± 16.03
Test statistic		0.21 (0.836)	0.35 (0.729)	−0.83 (0.407)	−0.36 (0.720)	0.01 (0.988)
**Help from friends**						
None	91 (42.33)	14.99 [14.04–15.94] ± 4.61	9.47 [8.39–10.55] ± 5.21	0.19 [0.046–0.35] ± 0.73	17.51 [16.18–18.83] ± 6.41	42.16 [39.54–44.79] ± 12.69
1–2	46 (21.40)	14.26 [12.59–15.93] ± 5.76	9.24 [7.74–10.73] ± 5.14	0.33 [−0.01-0.66] ± 1.16	17.11 [15.35–18.86] ± 6.04	40.93 [36.93–44.93] ± 13.77
3 +	78 (36.28)	13.59 [12.39–14.79] ± 5.36	10.88 [9.73–12.06] ± 5.15	0.32 [0.08–0.56] ± 1.08	19.52 [18.21–20.84] ± 5.91	44.32 [41.13–47.51] ± 14.32
Test statistic		1.56 (0.213)	2.09 (0.126)	0.44 (0.645)	3.11 (0.047)	1.02 (0.362)
**Help from family**						
None	91 (42.92)	14.99 [14.04–15.94] ± 4.61	9.47 [8.39–10.55] ± 5.21	0.19 [0.05–0.35] ± 0.73	17.51 [16.18–18.83] ± 6.41	42.16 [39.54–44.79] ± 12.69
1–2	46 (21.70)	14.26 [12.59–15.93] ± 5.76	9.24 [7.74–10.73] ± 5.14	0.33 [−0.01-0.66] ± 1.16	17.11 [15.35–18.86] ± 6.04	40.93 [36.93–44.94] ± 13.77
3 +	75 (35.38)	13.39 [12.18–14.59] ± 5.28	10.75 [9.58–11.92] ± 5.15	0.32 [0.07–0.57] ± 1.09	19.4 [18.06–20.74] ± 5.91	43.85 [40.60–47.11] ± 14.29
Test statistic		2.02 (0.136)	1.69 (0.1875)	0.43 (0.652)	2.69 (0.070)	0.71 (0.491)
**BMI**						
Underweight	4 (2.15)	19.75 [14.76–24.74] ± 5.06	11.75 [5.92–17.58] ± 5.91	0.5 [−0.49-1.49] ± 1	19.00 [14.97–23.02] ± 4.08	51.00 [43.32–58.68] ± 7.79
Normal	36 (19.35)	13.42 [11.99–14.84] ± 4.33	9.72 [8.72–11.46] ± 4.44	0.03 [−0.03-0.08] ± 0.17	17.86 [16.05–19.66] ± 5.49	41.03 [37.66–44.39] ± 10.23
Overweight	55 (29.57)	14.89 [13.51–16.28] ± 5.20	10.09 [8.73–11.46] ± 5.13	0.07 [−0.04-0.19] ± 0.42	18.98 [17.37–20.59] ± 6.06	44.04 [40.40–47.67] ± 13.67
Obesity	91 (48.92)	14.81 [13.73–15.89] ± 5.24	10.27 [9.11–11.43] ± 5.61	0.42 [0.16–0.67] ± 1.24	18.91 [17.55–20.26] ± 6.56	44.42 [41.44–47.39] ± 14.42
Test statistic		2.14 (0.970)	0.22 (0.880)	2.52 (0.060)	0.30 (0.828)	0.97 (0.408)
**Systolic**						
≤119	41 (18.89)	13.71 [12.13–15.28] ± 5.12	9.42 [7.95–10.88] ± 4.74	0.15 [−0.03-0.32] ± 0.57	17.27 [15.69–18.85] ± 5.14	40.54 [37.16–43.91] ± 10.97
120–139	83 (38.25)	14.90 [13.77–16.04] ± 5.23	10.89 [9.63–12.15] ± 5.83	0.34 [0.09–0.58] ± 1.12	19.13 [17.77–20.49] ± 6.28	45.27 [42.14–48.38] ± 14.42
140 +	93 (42.86)	13.99 [12.96–15.02] ± 5.04	9.62 [8.68–10.57] ± 4.63	0.28 [0.06–0.43] ± 0.92	18.09 [16.71–19.48] ± 6.76	41.96 [39.3–44.68] ± 13.35
Test statistic		1.02 (0.362)	1.74 (0.178)	0.58 (0.563)	1.32 (0.270)	2.17 (0.117)
**Diastolic**						
≤80	51 (23.50)	15.22 [13.83–16.60] ± 5.03	10.94 [9.49–12.39] ± 5.26	0.45 [0.11–0.79] ± 1.24	18.96 [17.47–20.46] ± 5.41	45.56 [42.10–49.03] ± 12.54
80–89	72 (33.18)	13.79 [12.55–15.03] ± 5.33	9.97 [8.72–11.23] ± 5.40	0.28 [0.03–0.52] ± 1.05	19.11 [17.52–20.70] ± 6.86	43.15 [39.67–46.63] ± 14.98
90 +	94 (43.32)	14.16 [13.14–15.18] ± 5.01	9.67 [8.67–10.67] ± 4.91	0.15 [0.02–0.28] ± 0.62	17.40 [16.13–18.68] ± 6.27	41.38 [38.83–43.93] ± 12.55
Test statistic		1.2 (0.302)	1.02 (0.362)	1.7 (0.185)	1.83 (0.163)	1.62 (0.199)

The overall coping mean (95%CI) ± SD score was 43.13 ± 13.57 and the scores for the individual domains involving; Spiritual, Collective, Ritual centered, and Cognitive/Emotional were 14.36 ± 5.14, 10.09 ± 5.18, 0.28 ± 0.96, and 18.40 ± 6.32, respectively (Table 1). For Resilience, the media score for relational and personal resilience were 32 (IQR-7) and 39 (IQR-9), respectively.

Ordinary Least Square regression analysis showed that the overall coping strategy was significantly influenced by systolic and diastolic pressure. Interestingly, having an increased systolic BP significantly increases the overall coping score compared with systolic BP < 120. Diastolic BP ranging from 90 or more significantly decreased overall coping by 6.05-unit score (95%CI = −11.94 to −0.17).

In the individual coping strategies domains, diastolic BP between 80 and 89 increased the unit score of spiritual coping by approximately 2.4 units (95%CI = 4.52 to −0.20) compared with diastolic BP < 80. With Collective coping strategy, having 3 or more friends significantly increase coping strategy by approximately 1.9 units significant (95%CI = 0.05–3.69) compared with patients with no friends. In addition, having a systolic BP ranges from 120 to 139 increased the Collective Coping by 2.9 unit score (95%CI = 0.58–5.31) compared with patients with < 120 systolic BP. Interestingly, no variable was associated with Ritual Coping strategy. For cognitive coping strategy, being aged 60–69 years significantly increased the strategy by 3.4 (95%CI = 0.00–6.79) compared with aged group < 39 years. Having 3 friends or more and increasing systolic BP significantly increase the log count of cognitive coping whiles diastolic BP of 90 and above significantly decrease the log count by 6.05 (95%CI = −11.94 to −0.17) (Table 2).

**TABLE 2:** Factors associated with overall coping strategies and the sub-domains among participants.

Variable	Spiritual	Collective	Ritual	Cognitive	Overall
	
	aβ [95%CI]	aβ [95%CI]	aβ [95%CI]	aβ [95%CI]	aβ [95%CI]
**Age**					
≤39	**Ref**	**Ref**	**Ref**	**Ref**	**Ref**
50–59	−1.38 [−4.25 to 1.49]	−0.26 [−3.45 to 2.94]	0.07 [−0.22 to 0.36]	2.75 [−0.46 to 5.96]	1.18 [−5.78 to 8.14]
60–69	−0.84 [−3.67 to 1.99]	0.45 [−2.76 to 3.67]	0.24 [−0.12 to 0.61]	3.39 [0.00–6.79]*	3.25 [−3.92 to 10.42]
70 +	−1.80 [−5.12 to 1.52]	0.41 [−3.08 to 3.90]	−0.11 [−0.46 to 0.24]	2.62 [−1.20 to 6.44]	1.12 [−7.06 to 9.30]
**Sex**					
Male	**Ref**	**Ref**	**Ref**	**Ref**	**Ref**
Female	1.70 [−0.07 to 3.48]	−0.06 [−2.04 to 1.93]	−0.24 [−0.55 to 0.08]	−0.21 [−2.15 to 1.74]	1.21 [−3.42 to 5.82]
**Marital status**					
Married	**Ref**	**Ref**	**Ref**	**Ref**	**Ref**
Single	−1.24[−5.04 to 2.55]	−0.89 [−4.79 to 2.99]	−0.22 [−0.57 to 0.12]	2.19 [−1.65 to 6.04]	−0.17 [−10.46 to 10.12]
Widowed	−0.27 [−2.17 to 1.63]	−0.26 [−2.33 to 1.82]	0.03 [−0.44 to 0.49]	1.22 [−1.20 to 3.65]	0.73 [−4.58 to 6.04]
Divorced	−2.08 [−5.08 to 0.92]	−2.97 [−5.11 to −0.82]**	−0.18 [−0.43 to 0.07]	−3.54 [−7.13 to 0.05]	−8.76 [−16.05 to -1.48]
**Educational level**					
No formal education	**Ref**	**Ref**	**Ref**	**Ref**	**Ref**
Primary/JHS	1.94 [−0.75 to 4.64]	3.52 [0.39 to 6.64]	−0.11 [−0.77 to 0.54]	−0.47 [−4.03 to 3.09]	4.89 [−2.36 to 12.14]
SHS/vocational	1.06 [−1.58 to 3.71]	1.76 [−1.42 to 4.93]	−0.06 [−0.58 to 0.46]	1.65 [−1.69 to 5.01]	4.41 [−2.84 to 11.66]
Tertiary	1.19 [−1.48 to 3.85]	1.28 [−1.89 to 4.46]	−0.33 [−0.85 to 0.18]	−0.54 [−4.22 to 3.13	1.59 [−5.65 to 8.84]
**Employment**					
Full time	**Ref**	**Ref**	**Ref**	**Ref**	**Ref**
Unemployed	−0.38 [−2.70 to 1.94]	−0.51 [−2.92 to 1.90]	0.23 [−0.15 to 0.62]	−0.95 [−3.55 to 1.65]	−1.61 [−7.69 to 4.48]
Retired	−0.97 [−3.16 to 1.21]	−1.22 [−3.44 to 0.99]	0.05 [−0.25 to 0.35]	−1.33 [−4.11 to 1.45]	−3.47 [−9.21 to 2.26]
**Religion**					
Christian	**Ref**	**Ref**	**Ref**	**Ref**	**Ref**
Islam	−1.82 [−6.10 to 2.45]	−0.97 [−5.36 to 3.46]	0.11 [−0.60 to 0.82]	−0.96 [−5.04 to 3.11]	−3.65 [−14.49 to 7.19]
**Help from friends**					
None	**Ref**	**Ref**	**Ref**	**Ref**	**Ref**
1–2	−0.91 [−3.27 to 1.46]	−0.85 [−3.04 to 1.33	0.16 [−0.16 to 0.48]	−0.04 [−2.29 to 2.21]	−1.64 [−7.00 to 3.72]
3 +	−1.26 [−2.86 to 0.34]	1.87 [0.05–3.69]*	0.18 [−0.17 to 0.54]	2.45 [0.31–4.59]**	3.24 [−1.32 to 7.79]
**BMI**					
Underweight	**Ref**	**Ref**	**Ref**	**Ref**	**Ref**
Normal	−4.43 [−10.24 to 0.79]	−1.79 [−7.83 to 4.25]	−0.38 [−1.36 to 0.59]	−1.85 [−7.69 to 3.99]	−8.76 [−19.74 to 2.23]
Overweight	−3.21 [−8.71 to 2.29]	−2.19 [−8.07 to 3.70]	−0.36 [−1.34 to 0.63]	−2.34 [−8.12 to 3.34]	−8.09 [−18.89 to 2.72
Obesity	−3.60 [−1.82 to 3.21]	−1.79 [−7.86 to 4.28]	0.13 [−0.92 to 0.97]	−1.616 [−7.56 to 4.33]	−6.88 [−17.91 to 4.15
**Systolic**					
<120	**Ref**	**Ref**	**Ref**	**Ref**	**Ref**
120–139	1.13 [−1.16 to 3.42]	2.94 [0.58–5.31]**	0.12 [−0.25 to 0.50]	3.12 [0.76–5.40]**	7.32 [1.56–13.07]*
140 +	0.69 [−1.82 to 3.21]	2.19 [−0.12 to 4.49]	0.45 [−0.07 to 0.97]	2.81 [0.37 to 5.26]*	6.14 [0.26–12.02]*
**Diastolic**					
<80	**Ref**	**Ref**	**Ref**	**Ref**	**Ref**
80–89	2.36 [−4.52 to −0.20]*	−1.29 [−3.49 to 0.90]	−0.26 [−0.081 to 0.28]	0.15 [−2.16 to 2.46]	3.78 [−9.25 to 1.69]
90 +	−1.81 [−4.29 to 0.66]	−1.92 [−4.47 to 0.63]	−0.59 [−1.26 to 0.09]	−1.74 [−4.02 to 0.55]	−6.05 [−11.94 to −0.17]*

aβ, adjusted coefficient estimate; ref, reference category. *P*-value notation: *p < 0.05, **p < 0.01.

Association between resilience and coping strategies were assessed using pairwise correlation and NBReg approach. For Personal Resilience (PR), there was positive and significant correlation with Collective, Cognitive, and overall coping strategies (*p*-value < 0.05). In addition, spiritual coping strategy, had a positive and significant correlation with relational resileince (RR). The regression analysis showed that, overall coping strategy significantly influenced PR and RR domains by 0.004 (95%CI = 0.002–0.01) and 0.005 (95%CI = 0.003–0.006) unit, respectively. Meanwhile, specific coping strategies showed that Collective and Cognitive debriefing significantly increase the unit of Resilience domains by approximately 0.01 (Table 3).

**TABLE 3:** The influence of coping strategies on resilience among the participants.

Coping strategy	Correlation analysis		OLS analysis	
		
	PR	RR	PR	RR
		
			aβ [95%CI]	aβ [95%CI]
Spiritual	0.096	0.14*	0.003 [−0.002 to 0.01]	0.005 [−0.00 to 0.01]
Collective	0.34***	0.42***	0.01 [0.001 to 0.01]***	0.01 [0.01–0.02]***
Ritual cantered	0.10	0.04	0.02 [−0.01 to 0.05]	0.008 [−0.02 to 0.03]
Cognitive debriefing	0.31***	0.29*	0.01 [0.003–0.01]***	0.007 [0.002–0.01]***
Overall	0.32***	0.35***	0.004 [0.002–0.01]***	0.005 [0.003–0.006]***

PR, personal resilience; RR, relational resilience; aβ, adjusted coefficient estimate; ref, reference category. *P*-value notation: *p < 0.05, ***p < 0.001.

Patients with controlled hypertension were observed to experience high level of coping strategies in all the coping domains including the overall coping score, however, this was not statistically significant compared with those with uncontrolled hypertension. In all the Resilience domains, there is a high level of resilience among patients whose hypertension was under control, compared to those with uncontrolled hypertension, although, not statistically significant (Tables 4, 5).

**TABLE 4:** The influence of coping strategies on control of hypertension.

Hypertension status	Spiritual	Collective	Ritual	Cognitive	Overall
	
	Mean [95%CI] ± SD	Mean [95%CI] ± SD	Mean [95%CI] ± SD	Mean [95%CI] ± SD	Mean [95%CI] ± SD
Uncontrolled	9.79 [8.93–10.65] ± 4.87	0.23 [0.09–0.39] ± 0.86	17.01 [16.87–19.14] ± 6.45	14.32 [13.41–15.23] ± 5.15	42.36 [40.02–44.70] ± 13.27
Controlled	10.47 [9.35–11.59] ± 5.56	0.33 [0.11–0.55] ± 1.08	18.92 [17.67–20.15] ± 6.14	14.42 [13.38–14.46] ± 5.16	44.14 [41.33–46.96] ± 13.95
Difference	−0.68	−0.09	−0.91	−0.11	−1.79
Interpretation	High among controlled	High among controlled	High among controlled	High among controlled	High among controlled
*T*-Test	−0.97	−0.71	−1.06	−0.15	−0.97

SD, standard deviation; Cl, confidence interval.

**TABLE 5:** Relationship between resilience and control of hypertension.

Hypertension status	Personal resilience	Relational resilience
	
	Mean [95%CI] ± SD	Mean [95%CI] ± SD
Uncontrolled	37.89 [36.63–39.15] ± 7.15	30.33 [29.29–31.37] ± 5.87
Controlled	39.12 [37.58–40.67] ± 7.67	30.45 [29.34–31.57] ± 5.54
Difference	−1.23	−0.12
Interpretation	High among controlled	High among controlled
*T*-Test	−1.24	−0.15

SD, standard deviation; Cl, confidence interval.

## Discussion

This study assessed the coping strategies and determined the relationship between coping and resilience among patients with hypertension. The overall coping score among study participants was 43.13 ± 13.57. This study showed that high systolic blood pressure had a positive significant relationship with overall coping score and cognitive domain score. Similarly, there was a significant positive relationship between high diastolic blood pressure and the spiritual coping domain. This implies that patients employ more spiritual coping strategies when diastolic blood pressure levels are high. This finding is in line with another study which found that religious or spiritual coping was associated with reduced risk of hypertension in African American women, especially among those reporting higher levels of stress (World Health Organization, 2012). This is not surprising given that Africans tend to rely on their faith to cope with serious illness (Kretchy et al., 2014). Again, social support with patients having 3 or more friends had high scores in the collective and cognitive coping domains. Overall coping had a positive and significant correlation with personal and RR, specifically collective and cognitive debriefing strategies significantly improved resilience among participants in this study.

Cognitive and emotional debriefing coping strategy was mostly used by patients while ritual-centered coping strategies were the least used in this study. Generally, patients with hypertension are found to use effective coping strategies when blood pressure is controlled and adopted avoidant or maladaptive coping strategies when blood pressures are high (Dolan et al., 1992; Casagrande et al., 2019). In line with this, our study showed that patients with controlled hypertension had high scores for coping strategies in all the coping domains compared to those with uncontrolled hypertension, although this association was not statistically significant. Contrary to this finding, in another study people with controlled hypertension did not make use of all the strategies in the coping domain as participants in this study made less use of task and emotion oriented coping strategies compared to those with uncontrolled hypertension (Awuah et al., 2019). In a study to assess the impact of coping responses to perceived racism on blood pressure levels in Black adults the regression analyses indicated that passive coping (i.e., avoidance) was associated with higher blood pressure levels and active coping was linked to lower blood pressure levels (Shah et al., 2022).

The current study also showed that high systolic blood pressure was positively associated with overall coping score and cognitive/emotional debriefing domains score. This implies that patients employed more cognitive/emotional debriefing coping strategies (active coping) in stressful situations where blood pressure is high. In contrast, while Emotion Oriented coping is supposed to reduce stress, it is not always successful and can actually induce stress and thus, indirectly raise blood pressure (Zapater-Fajarí et al., 2021). As a result, those who scored high on emotion-oriented coping might benefit from counseling therapies that rely on cognitive behavioral therapies as emotions may not be the best way to solve problems. Previous studies showed that, blacks who employed ineffective ways of coping were likely to develop hypertension (Malan et al., 2012; Venter et al., 2014).

Previous studies showed a significant and positive correlation between Emotion-oriented coping styles and blood pressure, while problem-oriented coping styles had a negative correlation with blood pressure (Saeed Jameshorani, 2022). Conversely, findings from another study observed a negative relationship between emotion-focused coping and systolic blood pressure levels among pregnant women (Chapuis-de-Andrade et al., 2022). They emphasized the need to reinforce the development of coping strategies which focus more on the problem than on the emotion, avoiding detrimental effects of emotional coping on blood pressure levels among pregnant women. The variations in the relationship between systolic blood pressure and coping strategies could be attributed to the different measures used in assessing coping strategies.

In addition, increasing age and having three friends or more increased the cognitive coping strategy. Social support and coping strategies are protective factors that serve as a buffer in stressful situations. In our study higher levels of social support (having 3 or more friends) improved patients’ use of cognitive coping strategies thereby improving their blood pressure management. In a study by Roohafza et al. (2014) looking at the role of perceived social support and coping styles individuals with depression and anxiety concluded that active coping styles such as cognitive coping strategy and perceived support are protective factors for psychological distress (Roohafza et al., 2014). Similarly, Aflakseir (2010) also demonstrated the protective role of social support in mental health and concluded that social support remains an important factor in managing chronic conditions.

This study found that decreased overall coping scores increased diastolic blood pressure. In a study that examined the effects of subjective stress and coping resources on blood pressure reactivity, there was a significant association between coping strategies and diastolic blood pressure and that the interactive effects of subjective stress and coping resources predicted diastolic blood pressure reactivity but not systolic blood pressure reactivity (Clark, 2003). These effects indicated that higher levels of problem-focused coping were related to more marked diastolic blood pressure changes during stressful situations and that emotion-focused coping was associated with less exaggerated diastolic blood pressure changes during less stressful situations. In another study, more active coping strategies were associated with higher diastolic blood pressure (Wright et al., 2020). These findings highlight the potential contribution of coping to the management of hypertension among Ghanaians.

Furthermore, our study found that increased diastolic pressure was associated with increased Spiritual coping. Our study also showed high scores in the Spiritual coping domain among patients with controlled hypertension. Religiosity and spirituality have long been associated with hypertension. Buck et al. (2009) reported similar findings in their study that examined the relationship between multiple dimensions of religiosity, blood pressure, and hypertension. They indicated that prayer was associated with an increased likelihood of hypertension and spirituality was associated with increased diastolic blood pressure. Similarly, another study that assessed blood pressure, depression, and coping among different cultures noted that Blacks used ritual and spiritual coping strategies whiles Whites consistently used spiritual coping to deal with their depression and chronic hypertensive status (Le Roux et al., 2018). Thus, patients in our study were more inclined to resort to spiritual understandings when diastolic pressure increases rather than resorting to cognitive and emotional debriefing in dealing with stressful situations. It is unclear why spirituality is associated with increased diastolic blood pressure.

Again, our study showed that an increased systolic BP was associated with increased use of collective coping and that having 3 or more friends also significantly increased collective coping strategy compared to patients with no friends. This implies that patients in our study sought more social support to cope with high systolic pressure. This contradicts the study by Le Roux et al. (2018) which reported utilizing more avoidance or loss-of-control coping and seeking less social support or isolation as a coping mechanism. These findings highlight the need to emphasize the beneficial effect of social support in hypertension management.

In terms of Resilience, the study observed a significant correlation between overall coping and three components of coping: collective coping, cognitive and emotional debriefing, and spiritual coping. The regression analysis showed that, PR positively correlated with collective, cognitive, and emotional debriefing, and overall coping strategies. Similarly, RR positively correlated with the spiritual coping domain. Again, in the regression analysis, overall coping had a positive and significant correlation with personal and RR, specifically collective and cognitive debriefing strategies significantly improved resilience among hypertensive patients in this study. The findings of this current study are novel as previous studies, to the best of our knowledge have not explored the relationship between resilience and coping particularly among patients with hypertension. However, it is not surprising as other studies have found that the positive contribution of higher levels of resilience and an adaptive coping strategy improves the level of health of patients with chronic diseases (Cal et al., 2015; Ghanei Gheshlagh et al., 2016; Li and Miller, 2017; Popa-Velea et al., 2017; Macía et al., 2021; Zapater-Fajarí et al., 2021).

This study focused on one chronic disease (hypertension), thus the findings cannot be generalized to other chronic diseases such as asthma, sickle cell disease, cancer, and diabetes. Future studies should fill this gap by involving other chronic illnesses to understand the effect of coping and resilience in people with chronic diseases more holistically. In view of the cross-sectional nature of this study, the associations cannot be used to demonstrate causality and the results of the coping strategies should be viewed in that context.

## Conclusion

This study demonstrated that cognitive and emotional debriefing coping strategy was mostly used by patients and high systolic blood pressure was significantly associated with overall coping score and cognitive/emotional debriefing domain scores. In addition, increasing age and having social support increased cognitive coping strategy. Coping had a positive and significant correlation with personal and RR, specifically collective and cognitive debriefing strategies significantly improved resilience among study participants. This is an important finding as it demonstrates the need to introduce measures to improve the coping mechanisms and resilience of patients to help with effective management of hypertension.

## Data availability statement

The raw data supporting the conclusions of this article will be made available by the authors, without undue reservation.

## Ethics statement

The protocol was approved by the University of Ghana College of Health Sciences Ethical and Protocol Review Committee and the protocol identification number was CHS-Et/M.4-P4.1/2020-2021. The patients/participants provided their written informed consent to participate in this study.

## Author contributions

VB, EY, and VG contributed to the concept, design, conduct, and manuscript writing. AG-M and GE-F involved in the design, conduct, and drafting of the manuscript while IK and CM-K helped with writing of manuscript and the final editing. All authors contributed to the article and approved the submitted version.
